# The bald and the beautiful: hairlessness in domestic dog breeds

**DOI:** 10.1098/rstb.2015.0488

**Published:** 2017-02-05

**Authors:** Heidi G. Parker, Alexander Harris, Dayna L. Dreger, Brian W. Davis, Elaine A. Ostrander

**Affiliations:** National Human Genome Research Institute, National Institutes of Health, Bethesda, MD 20892, USA

**Keywords:** canine, genomics, mutation, breed, variation, domestication

## Abstract

An extraordinary amount of genomic variation is contained within the chromosomes of domestic dogs, manifesting as dramatic differences in morphology, behaviour and disease susceptibility. Morphology, in particular, has been a topic of enormous interest as biologists struggle to understand the small window of dog domestication from wolves, and the division of dogs into pure breeding, closed populations termed breeds. Many traits related to morphology, including body size, leg length and skull shape, have been under selection as part of the standard descriptions for the nearly 400 breeds recognized worldwide. Just as important, however, are the minor traits that have undergone selection by fanciers and breeders to define dogs of a particular appearance, such as tail length, ear position, back arch and variation in fur (pelage) growth patterns. In this paper, we both review and present new data for traits associated with pelage including fur length, curl, growth, shedding and even the presence or absence of fur. Finally, we report the discovery of a new gene associated with the absence of coat in the American Hairless Terrier breed.

This article is part of the themed issue ‘Evo-devo in the genomics era, and the origins of morphological diversity’.

## 1. Background

Domestic dogs are unique among land mammals in that they display an extraordinary amount of phenotypic variation across populations or breeds [1,2]. Over 400 breeds are recognized worldwide, with over 170 recognized by the American Kennel Club (AKC) [3]. Dog breeds have established a unique niche in human genetics because of the extraordinary parallels that exist between human and canine diseases [4–6]. In this paper, we focus on canine morphology, specifically related to pelage.

It is well established that dogs of a particular breed share an extraordinary similarity in terms of appearance and growth rate due to strong selection for specific traits [7–10]. Such selection has gone on, in some cases, for hundreds of years. One of the unique advantages of domestic dogs for mapping traits has been the availability of large families, with single stud dogs often producing dozens of litters, as well as the rigour with which breed structures are maintained [7]. Such resources have been used to understand the genetic underpinning of body size, leg length, coat colour, skull shape and others (reviewed in [11,12]), with genome-wide studies highlighting the many regions under selection in dog breeds [8–10,13]. The reduction in genetic diversity among purebred dogs either as a result of breeding trends such as line-breeding and the use of popular sires, or population bottlenecks stemming from breed formation, has reduced the level of heterogeneity and increased the extent of linkage disequilibrium across the genome, making the task of finding significantly associated loci easier than similar studies in humans [7,14].

**Figure 1.**
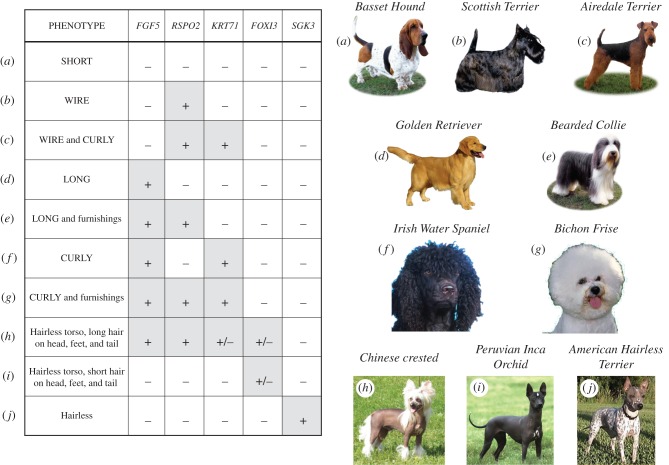
Combinations of alleles produce distinctly different pelage phenotypes. Combinations of alleles at five genes—*RSPO2*, which controls fur growth pattern or furnishings; *FGF5*, which controls much of the fur length phenotype, and *KRT71*, which contributes to curl; and *FOXI3* and *SGK3,* which produce hairlessness—are shown. Dogs showing the distinct phenotypes are presented to the right with letters (*a*)–(*j*) corresponding to the combinations of genotypes on the left. Revised from [15].

We, and others, have undertaken studies to understand the selection for various fur types in dog breeds. While not all aspects of fur have been defined, length, curl, growth pattern, shedding and hairlessness are among those that have been associated with specific mutations (figure 1). We review current thinking regarding each of these and present new data on hairlessness in dogs.

## 2. Fur growth pattern, curl and length as breed traits

For many dog breeds the length, degree of curl and hair growth pattern are a key part of the breed standard. For each trait, some breeds adopt rigorous standards, while others allow a small amount of variation.

*R-spondin 2* (*RSPO2*) is one of three genes we identified in 2009 that contribute enormously to the overall appearance of dogs [15]. ‘Furnishings’ is a term that refers to the presence of coarse fur growth in a pattern that resembles a moustache and eyebrows, such as in the Schnauzer and Scottish Terrier (figure 1*b*). This pattern corresponds to a coat type called wire-haired in many otherwise short-haired breeds of dog describing a phenotype of coarse, bent guard hairs that stick out away from the body giving a wiry appearance. To identify the underlying mutation, we first undertook a genome-wide association study (GWAS) of 903 dogs from 80 domestic breeds, using a panel of single-nucleotide polymorphisms (SNPs) that produced data on approximately 41 000 usable SNPS, and which spanned the dogs' 38 autosomes and X chromosome. We verified our findings in a single breed, the Dachshund, with and without furnishings. After analysis and fine mapping, the underlying mutation was found to be an insertion of 167 bp in the 3′ non-coding region of the *RSPO2* gene (*p* = 10^−241^). The trait is under strong selection within a number of breeds and probably affects mRNA stability.

The *RSPO2* gene interacts with the *Wnt* gene to activate B-catenin [16]. *Wnt* signalling is required for hair follicle development [17] and the *Wnt-B-catenin* pathway is critical in the development of hair follicle tumours in humans [18]. Such tumours are reportedly most common in dogs with furnishings [19]. Together, these facts make a strong case that *RSPO2* is indeed responsible for the phenotype of furnishings, a result that has since been replicated [8–10].

For each fur-related trait we mapped, we used a similar strategy as above [15] ruling out false positives due to population substructure, a common problem in dog genetic studies, and performing a second validation using a unique dataset. Finally, we undertook fine mapping of each primary locus to find a haplotype defining the critical gene and eventually a mutation. This strategy was used to find a gene underlying fur length which we now know is controlled, at least in part, by one or more variants in the *fibroblast growth factor 5* (*FGF5*) gene, consistent with previous reports in mice [20] and a locus on chromosome 1 that includes the *melanocortin 5 receptor* (*MC5R*) [10]. Finally, tight versus loose curl is caused by a single nucleotide variant in *keratin 71* (*KRT71*) [15]. The latter finding was consistent with mouse studies, which identify the reduced coat 12 mutation (Rco12) in the *KRT71* gene, which is important in bending of the hair shaft [21].

The results of the GWAS for length and curl were overwhelmingly significant (*p* = 10^−157^ and 10^−92^, respectively), though not as strong as we observed in furnishings, probably because there is redundancy in the pathways that lead to these phenotypes. In aggregate, these three genes have high predictive value for what a dog's coat will look like (figure 1). Note that not all phenotypic combinations are seen. For instance, a breed cannot be curly coated, no matter what the *KRT71* genotype if the hair is very short, as the fur is simply not long enough to curl, and interestingly, we do not find this genotype combination. Findings regarding each of these genes have been confirmed in independent studies [8–10].

## 3. Other fur characteristics are controlled by a small number of genes

Additional fur-related traits are clearly under selection in a subset of breeds, as shown by other studies, some of which relate to data from humans [9]. For instance, the *MC5R* gene, which has been associated with hair length in some breeds, is expressed in sebaceous glands in humans [22]. Likewise, mice deficient in the gene suffer from a severe defect in water repulsion and thermoregulation, caused by a decreased production of sebaceous lipids [23]. These findings hint that mutations in this gene could be related to thermoregulation via water repulsion of the dog coat [10], and it would be interesting to see if the mutation observed in this study, which purportedly causes a conformation change in the encoded protein structure, is more strongly associated with breeds that are ‘water friendly’, including the Newfoundland dog, Portuguese Water Dog (PWD) and the retriever breeds (Chesapeake, Golden, Labrador), versus those that are water adverse, such as the Sheltie and some of the Spitz-type breeds.

*MC5R* and *RSPO2* may, together, affect shedding in dogs. In a study of PWD, a breed that does not typically shed, it was noted that dogs that did not carry the *RSPO2* mutation did shed their coat [24]. Indeed, breeds that rarely shed, such as the poodle, are almost always homozygous for the derived allele in *RSPO2.* Heavy-shedding breeds, or those breeds that shed and have a heavy undercoat, tend to carry the ancestral allele at *MC5R* [10].

## 4. Hairless dogs

Hairless dogs across the world have been recognized since the time of Darwin, who wrote about naked Turkish dogs with defective teeth ([25,26] and references within). While many hairless dogs have reportedly existed over time, many are now extinct and no more than half a dozen are recognized around the world today. The best recognized in North America are the American Hairless Terrier (AHT), Chinese crested dog and the Mexican Hairless Dog, now known as the Xoloitzcuintli, all of which are recognized by the AKC, as is the Peruvian Inca Orchid, which is derived from the Peruvian Hairless Dog. Prized by the early explorers and carried on ships to contain the rat population, hairless dogs came in many sizes. In the US, the smallest variety of the Xolo was called the Mexican Hairless Dog up until the 1960s [27]. For our purposes here, we refer to all varieties as the Xolo. Other hairless breeds include the Argentine Pila dog, the Ecuadorian Hairless dog, Abyssinian Sand Terriers, the African hairless and the Hairless Khala also from Argentina. Many of these breeds are very rare and the latter are not recognized by any breed registry [28,29].

### (a) Chinese crested, Xolo and Peruvian Hairless breeds

Among the most interesting of the hairless dogs is the Chinese crested dog, a breed for which multiple varieties exist. The most recognizable is the hairless Chinese crested, whose hairlessness is categorized as a form of canine ectodermal dysplasia (CED) [30], with silky tufts of hair present only on the dome of the head, extremities and tail. These dogs have additional health issues, including defects in teeth, nails, sweat glands, etc. [31,32]. The overall phenotype is similar to what is observed in the Xolo and Peruvian Inca Orchid; however, these breeds often lack the long hair tufts of the Chinese crested.

Inherited as a single gene semi-dominant trait, the CED locus was initially mapped to canine chromosome 17 using microsatellites in 2005 [33]. After excluding candidate genes such as *ectodysplasin A1 receptor* (*EDAR*) [33,34], a GWAS and additional fine mapping showed that CED was due to a mutation in the *forkhead box transcription factor* (*FOXI3*) [35]. This gene, which is a member of a large transcription factor family, is specifically expressed in developing hair and teeth, and is a regulator of ectodermal development [32], making it a superb candidate for the trait [35]. The underlying mutation is a 7 bp duplication in exon 1 of the gene, yielding a frameshift that produces a premature stop codon and loss of 95% of the normal protein. In addition to the Chinese crested, the Xolo and Peruvian Hairless Dog carry the same mutation [35]. The lack of homozygotes in either breed suggests that the mutation is embryonic lethal, perhaps due to other functions of the gene.

The fact that these hairless breeds all share the exact mutation is not surprising despite their reportedly diverse origins. As a dominant trait the novel hairless phenotype would have been easy to perpetuate in any breed in which it was crossed. There is little verified history of the breeds independently, as both the Chinese and the South American dogs have been reported to be in the Americas prior to European colonization [36]. Whether this is an indication of early trade between Asia and the new world, prehistoric migration from Asia across the Bering Strait, or a lack of distinction between the breeds in early days is unknown. The Xolo purportedly existed in Mexico at least since the time of the Aztecs and were at one time considered sacred, as the dogs were thought to be needed by their deceased master to guide their souls through the afterlife [31]. Similarly, the Peruvian Hairless Dogs are reported to have been present during the time of the Incan Empire [29]. By comparison, the Chinese crested may have descended from African hairless dogs or vice versa [37,38]. Regardless of their origins and which came first, a lack of standardized breeding practices in the early 1900s blurred the lines between these breeds leading to their removal from AKC registries for more than two decades and the more recent revival of the breeds in Europe and Mexico [29]. A SNP haplotype spanning 102 kb shows that the *FOXI3* mutation in all three breeds came from the same ancestral source. It is difficult to say which breed first originated the mutation because they were derived from or crossed with each other prior to breed establishment.

The hairless Chinese crested dogs can show varying degrees of hairlessness from true hairless to semi-coated [37], suggesting that there may be modifier genes for the trait. Because this form of hairlessness is by necessity heterozygous, there are always coated dogs born as littermates to the hairless pups. This variety is known as ‘powderpuff’ in the Chinese crested. Studies aimed at understanding the phenotypic differences in hair between the hairless Chinese crested and powderpuffs report that the hairless dogs have only simple primary hair follicles compared with the compound follicles of the powderpuffs and other coated breeds [39,40]. Based on these findings in dogs, the role of *FOXI3* in hair follicle development has been further studied in mouse models where it has been shown to play a role in stem cell activation affecting post-natal hair growth and hair regeneration [41]. In addition to hairlessness, the dogs with FOXI3 mutations often have problems with dentition, such as missing teeth, and less commonly, malformations of the ear. This latter, secondary phenotype led to the identification of a rare human mutation involving the deletion of the *FOXI3* gene in a child with microtia, an underdeveloped outer ear [42]. This remains an interesting case where a naturally occurring mutation has been under strong selection to retain only one part of the phenotype in dogs, yet can improve our knowledge of the role of the gene in development as well as contribute to our understanding of rare human disorders.

### (b) American Hairless Terrier

**Figure 2.**
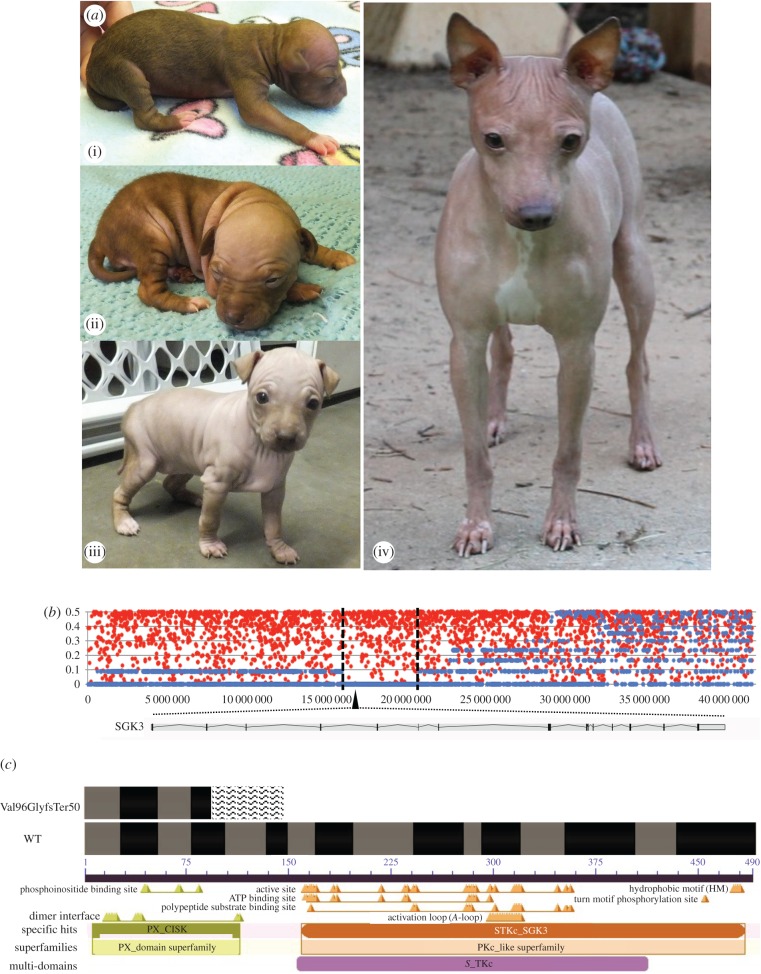
Homozygosity due to strong selective pressure identifies a deletion in *SGK3* that causes the hairless trait found in the AHT. (*a*) AHTs are born with sparse fur and quickly lose it over the first few weeks after birth. Images (i) newborn; (ii) two weeks; (iii) five weeks; (iv) adult. (*b*) Pattern of heterozygosity (*H*_e_) over chromosome 29 in AHT (blue) and 858 dogs from 89 breeds (red). SNP positions along chromosome 29 from the centromere to the telomere are on the *x*-axis, *H*_e_ is on the *y*-axis. The boundaries of the homozygous region are indicated with dotted lines and the position of the SGK3 gene is indicated by the black triangle. (*c*) Schematic of the SGK3 gene with and without the AHT mutation. Black and grey bars in the genes denote the exons as predicted by Ensembl [44]. The wavy lines indicate the predicted nonsense sequence produced by the frame shift. Putative active sites and protein domains are shown below the gene. The protein domains are predicted by NCBI-conserved domain database [45]. Photographs of the AHT provided by Teri Murphy.

The AHT is the only hairless breed in which the trait is recessive [43], and therefore, the homozygous state is not lethal. Indeed, the AHT are remarkably healthy dogs born with a sparse, fuzzy coat that is lost completely within the first months (figure 2*a*). The Rat Terrier breed, the predecessor of the AHT, originated with mixed-breed terriers called Feists that came from Europe to North American in the eighteenth century. The modern Rat Terrier was produced from that population following addition of the Beagle, Miniature Pinscher and Italian Greyhound breeds. The Rat Terrier was recognized as a distinct breed by the United Kennel Club in 2004 and the AKC in 2013.

**Figure 3.**
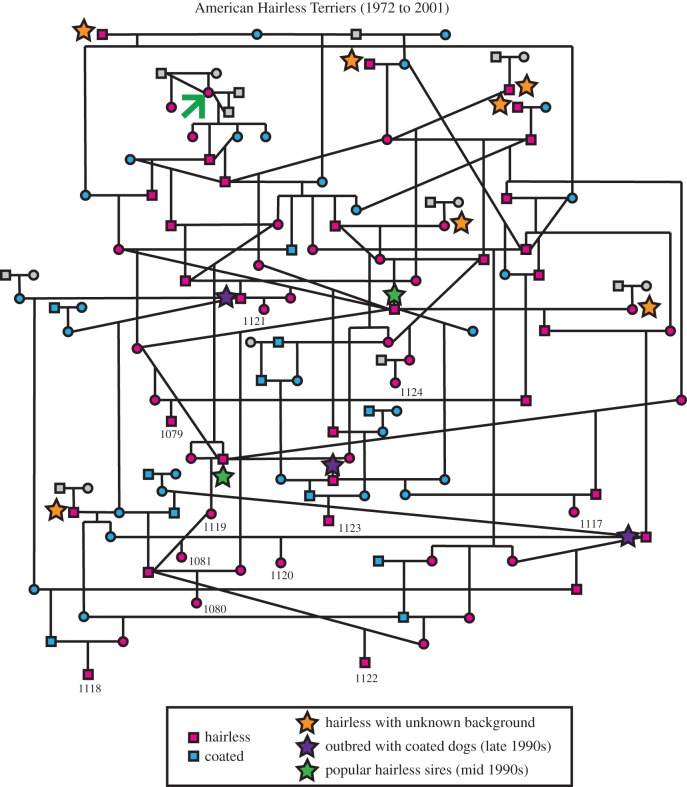
Pedigree of the AHT from 1972 to the present day. The first AHT (green arrow) was the offspring of two coated Rat Terriers. The hairless trait (pink) was captured through generations of careful backcrossing, after which unrelated coated Rat Terriers (blue) were included in the gene pool, particularly in the mid-to-late 1990s (outcrosses indicated by purple stars). The AHT club of America indicates that there had been other hairless Rat Terriers produced prior to the AHT founder (AHTCA, http://www.ahtca.info/index.html) and this pedigree shows the inclusion of a few hairless individuals without known family connections to the founder (orange stars). Green stars indicate popular sires within the breed. The dogs used in this study are indicated by a number under the symbol.

It is believed that in 1972 a breeding of Rat Terriers produced a hairless and generally attractive puppy that was otherwise lively and healthy. This was not the first time a Rat Terrier had been born hairless but in this instance the owners recognized her unique features and carefully bred her to selected individuals to produce today's AHT (figure 3). Owing to their heritage, the AHT is strikingly similar in appearance to its ancestor, the Rat Terrier, and was considered a variety of Rat Terrier for many years. Therefore, once true-breeding of the hairless phenotype was achieved, coated Rat Terriers were crossed back into the lines to maintain and increase the healthy gene pool for the AHT while selecting for the hairless trait [46] (figure 3). The AHT is the newest AKC-recognized breed, having achieved such status in 2016.

To identify the mutation in the AHT, in accordance with the NHGRI Institutional Animal Care and Use Committee (IACUC), we first undertook SNP chip and homozygosity studies. We decided to forego GWAS because of the high chance of false positives stemming from the close familial relationships between the hairless dogs and a lack of coated dogs sharing that family structure. We started with DNA samples from 11 hairless AHTs, indicated by numbers on the pedigree in figure 3, which we ran on the Illumina Canine HD SNP chip (Illumina, San Diego, CA), using standard protocols. Genotype calls from 161 151 informative SNPs were made with Genome Studio V2011.1 with genotyping module v. 1.9.4 (Illumina). Each SNP was analysed for homozygosity within all hairless dogs (*H*_e_ = 0) and total number of contiguous SNPs was tallied, as well as total length of each homozygous region. This revealed a region of 363 contiguous SNPs on chromosome 29 that were homozygous in all 11 hairless AHTs (figure 2*b*). These SNPs spanned greater than 4.8 Mb from chr29 : 15973319–20794824 in the CanFam3.1 assembly. The second longest run of homozygous SNPs comprised only 71 SNPs on chr15. The 4.8 Mb region on chr29 included 25 putative protein-coding genes.

We next sequenced the entire genome of a single hairless AHT using protocols we described previously [47]. The vcftools command vcf-contrast [48] was used to identify genotypes found in the AHT that were not found in 89 other dogs with whole genome sequence data. A list of the sequenced breeds and their accession numbers can be found in electronic supplementary material, table S1. The predicted effects of all identified SNPs and indels were determined using snpEFF [49] and the Ensembl canine gene model CanFam3.1.76 [44].

Whole genome sequence of a single AHT revealed 3994 variants that were homozygous in the AHT and not found in any of 89 other dogs with complete whole genome sequence available (electronic supplementary material, table S1). Of these, 24 were predicted to alter a protein; 21 missense mutations, two in-frame insertions and one frameshift deletion. Only one, the frameshift deletion, was located within the 4.8 Mb homozygous region on chr29. The deletion removes four bases (TTAG) from chr29 : 16366702–16366705 within exon 4 of the *serum/glucocorticoid regulated kinase family member 3* gene (*SGK3*). This deletion alters the reading frame of the protein at amino acid 96 creating a new protein sequence for 50 amino acids and a premature stop at amino acid 157. This mutation is predicted to knock out the original function of the gene as it removes the entire STKc_SGK3 catalytic domain for which the gene is named (figure 2*c*). *SGK3* has been shown to be important in post-natal hair follicle development in studies of mice [50–52]. This is particularly applicable to the canine condition as the AHT are born with hair, which is lost over the first two months after birth (figure 2*a*).

Next, the 4 bp deletion found at chr29 : 16366702 was resequenced in 12 hairless AHT and four coated Rat Terriers using the primers F-GTACATCAAGAAACATGAATTAAGAAA, R-GCACAGTAACATTCCACAGAACA and F-CGATCAAACTTCACTGTCT, R-ATGAGTTGATGGAGGGAAA, with BigDye v. 3.1 and an ABI3730*xL* DNA analyser (Thermo Fisher Scientific). All hairless AHT were found to have two copies of the mutation, while the four coated Rat Terriers were wild-type at both alleles.

## 5. Summary

With the dozens of phenotypic traits that define them, modern domestic dog breeds are a self-contained study in genetics. Analyses of many phenotypes, for instance body size [8,10,53], have shown that subtle changes in small numbers of genes result in large phenotypic differences [5]. This is not surprising as dogs are presumed to have been domesticated from wolves only about 15 000 years ago and most breeds were defined in the last few hundreds of years [54–56]. From the viewpoint of genetics, this offers a fantastic opportunity to identify genes and the parts of those genes that control various phenotypes that are of interest to human geneticists, developmental biologists and those studying both rare and common diseases. In this study, we focused on the simple phenotype of dog fur leaving aside the issue of coat colour. Defining phenotypes such as length, curl, growth pattern, shedding and the presence or absence of fur, we show how a small number of genes contribute to these identifiable differences in breed appearance. Hairlessness is of particular interest as a small number of breeds from seemingly disparate regions of the earth share common mutations. In this paper, we report a distinctly new gene and mutation, SGK3^Val96GlyfsTer50^, that causes hairlessness in a recently developed breed, the AHT. This result, plus the others summarized here, highlight the relevance, and to some degree the roles, of specific genes in hair development.

Key to every successful genotyping study is strong phenotypes. In the canine system, those are provided in the form of breeds, of which hundreds exist with clear descriptions. As shown here with the AHT, more breeds are created frequently in response to dog fanciers' wishes. Current and future breeds offer ever more chances to find genes that provide insight into our history and development, as well as pieces of a genetic puzzle to which all mammals belong.

## Supplementary Material

Supplementary Table 1

## Data accessibility

All whole genome sequences have been submitted to the SRA and NCBI. The accession numbers for all sequences referred to in this manuscript are given in electronic supplementary material, table S1.
